# Role of Functional Neuroimaging with 123I-MIBG and 123I-FP-CIT in De Novo Parkinson’s Disease: A Multicenter Study

**DOI:** 10.3390/life13081786

**Published:** 2023-08-21

**Authors:** Maria Silvia De Feo, Viviana Frantellizzi, Nicoletta Locuratolo, Arianna Di Rocco, Alessio Farcomeni, Caterina Pauletti, Andrea Marongiu, Julia Lazri, Susanna Nuvoli, Francesco Fattapposta, Giuseppe De Vincentis, Angela Spanu

**Affiliations:** 1 Department of Radiological Sciences, Oncology and Anatomo-Pathology, Sapienza, University of Rome, 00161 Rome, Italyj.lazri@yahoo.it (J.L.);; 2 Department of Human Neurosciences, Sapienza, University of Rome, 00161 Rome, Italy; 3 National Centre for Disease Prevention and Health Promotion, National Institute of Health, 00161 Rome, Italy; 4 Department of Economics & Finance, University of Rome “Tor Vergata”, 00133 Rome, Italy; 5 Department of Medical, Surgical and Experimental Sciences, University of Sassari, 07100 Sassari, Italy

**Keywords:** 123I-MIBG, 123I-FP-CIT, Parkinson’s disease

## Abstract

Background: Parkinson’s disease is a progressive neurodegenerative disorder, with incidence and prevalence rates of 8–18 per 100,000 people per year and 0.3–1%, respectively. As parkinsonian symptoms do not appear until approximately 50–60% of the nigral DA-releasing neurons have been lost, the impact of routine structural imaging findings is minimal at early stages, making Parkinson’s disease an ideal condition for the application of functional imaging techniques. The aim of this multicenter study is to assess whether 123I-FP-CIT (DAT-SPECT), 123I-MIBG (mIBG-scintigraphy) or an association of both exams presents the highest diagnostic accuracy in de novo PD patients. Methods: 288 consecutive patients with suspected diagnoses of Parkinson’s disease or non- Parkinson’s disease syndromes were analyzed in the present Italian multicenter retrospective study. All subjects were de novo, drug-naive patients and met the inclusion criteria of having undergone both DAT-SPECT and mIBG-scintigraphy within one month of each other. Results: The univariate analysis including age and both mIBG-SPECT and DAT-SPECT parameters showed that the only significant values for predicting Parkinson’s disease in our population were eH/M, lH/M, ESS and LSS obtained from mIBG-scintigraphy (*p* < 0.001). Conclusions: mIBG-scintigraphy shows higher diagnostic accuracy in de novo Parkinson’s disease patients than DAT-SPECT, so given the superiority of the MIBG study, the combined use of both exams does not appear to be mandatory in the early phase of Parkinson’s disease.

## 1. Introduction

Parkinson’s disease (PD) is a progressive neurodegenerative disorder with incidence and prevalence rates of 8–18 per 100,000 people per year and 0.3–1%, respectively [1,2,3]. The onset of PD before the age of 50 years is rare, with a significant increase in incidence after the age of 60 years [4]. It is currently believed that a combination of interacting genetic changes and environmental factors may be responsible for this condition [5]. Even though the main pathological characteristic of PD is the progressive loss of dopamine (DA)-releasing neurons in the substantia nigra associated with the presence of cytoplasmic inclusions known as “Lewy bodies” [6], pathological processes in PD are not limited within the dopaminergic system, but several other systems, such as the serotonergic, glutamatergic, opioid, noradrenergic and cholinergic systems, are involved [7].

### 1.1. Clinical Manifestations and Diagnosis

The disease is classically accounted among movement disorders in relation to its characteristic motor phenomenology. The diagnosis of PD in the living patient is currently clinical, based on the presence of the cardinal motor signs that are core features of the disease, such as bradykinesia associated with rest tremor or rigidity and postural imbalance later in the course of the disease [8]. PD is actually a complex condition with a wide range of non-motor symptoms, such as as cognitive and behavioral disorders, genitourinary and gastrointestinal problems and fatigue, which contribute to severe disability not only in the advanced stage; olfactory deficit, constipation, rapid-eye movement, REM-behavior disorder (RBD) and depression may precede the appearance of motor symptoms by more than a decade. The appearance of these symptoms is closely related with the progression of Lewy pathology in PD and may be driven by deficiencies in non-dopaminergic neurotransmitters or by an overlap between dopaminergic and non-dopaminergic systems [9]. As parkinsonian symptoms do not appear until approximately 50–60% of the nigral DA-releasing neurons have been lost, the impact of routine structural imaging findings is minimal at this stage, making PD an ideal condition for the application of functional imaging techniques [10].

### 1.2. Imaging Techniques

Due to the widespread availability of single-photon emission computed tomography (SPECT) scans and approval from European and US medical agencies, SPECT imaging with the 123I-labeled cocaine analogue “N-ω-fluoropropyl-2β-carbomethoxy-3β [1] nortropane” (FP-CIT) is widely used in clinical practice [11]. The radiopharmaceutical binds with high affinity to the dopamine transporter (DAT) located on presynaptic terminals of dopaminergic neurons, thus identifying the loss of functional dopaminergic neuron terminals of the nigrostriatal pathway [1,12]. FP-CIT (DAT)-SPECT studies have demonstrated high sensitivity (87–98%) and specificity (80–100%) in differentiating patients with PD (reduced presynaptic uptake) from both controls and patients with essential tremor or non-degenerative vascular and drug-induced parkinsonism (normal uptake) [13]. However, 123I-FP-CIT cannot be used to differentiate PD from atypical forms of neurodegenerative parkinsonian syndromes (non-PD) such as progressive supranuclear palsy (PSP), multiple system atrophy (MSA) and corticobasal syndrome (CBS).

In this regard, the differential clinical diagnosis principally with respect to the MSA-parkinsonism (MSA-P) phenotype and the variants of PSP known as PSP-Richardson syndrome (PSP-RS) and PSP-P (PSP-predominant parkinsonism), the latter being observed in up to 35% of cases, is particularly challenging because of the overlapping in the early clinical picture [14]. Recently, Alster and colleagues explored the potential role of perfusion SPECT in differentiating PSP-P and MSA-P in patients with a 3–6-year history of disease duration with interesting results [15]. They revealed significant differences between PSP-P and MSA-P in the frontal lobe, with more pronounced hypoperfusion in this region observed in PSP-P [16].

Myocardial scintigraphy with the 123I-labeled guanethidine analog “metaiodobenzylguanidine” (mIBG-scintigraphy) can be used to assess the integrity of presynaptic postganglionic sympathetic fibers [17], typically damaged in alpha-synucleinopathies such as PD and LBD [18,19] but preserved or only mildly reduced in CBD, PSP and MSA [8], thus allowing for differential diagnosis [20,21]. A 2012 systematic review evidenced that the pooled sensitivity of mIBG-scintigraphy in detecting PD was 88%, while the pooled specificity in discriminating between PD and non-PD was 85% [22]. Despite their increased use in clinical practice for both diagnosis and differential diagnosis, the role of these functional imaging techniques in patients with recent onset of suspected symptoms has not yet been clarified.

The aim of this multicenter retrospective study involving two different Italian centers is to assess whether DAT-SPECT, mIBG-scintigraphy or an association of both exams presents the highest diagnostic accuracy in such de novo PD patients.

## 2. Materials and Methods

The study was performed in accordance with the ethical standards of the 1964 Declaration of Helsinki and its later amendments.

A total of 288 consecutive patients with suspected diagnoses of PD or non-PD syndromes were analyzed in the present Italian multicenter retrospective study. All subjects were de novo, drug-naive patients and met the inclusion criteria of having undergone both DAT-SPECT and mIBG-scintigraphy within one month of each other. Imaging studies were performed in two different Italian centers.

Diagnosis of idiopathic PD according to the International Parkinson and Movement Disorder Society (MDS) clinical diagnostic criteria for PD (Postuma Mov Disord 2015) was independently performed and confirmed by at least two neurologists experienced in movement disorders. Patients with PD were classified into tremor dominant (TD), akinetic-rigid (AR) or mixed type according to the clinical phenotype at disease onset. Hoehn and Yahr stages (H&Y) range from I to III.

Non-PD was diagnosed in patients with clinical criteria of PSP [23,24], LBD [25] and MSA [26]. Patients were investigated with regard to neurological family history and the presence of other related neurologic and cardiologic diseases. Drugs that may interact with DAT-SPECT results, including amoxapine, amphetamine, benztropine, bupropion, escitalopram, cocaine, mazindol, methamphetamine, methylphenidate, norephedrine, phentermine, phenylpropanolamine, sertraline and paroxetine, were recorded. Metabolic disorders, diabetes and treatments affecting one or both 123I-FP-CIT and 123I-mIBG uptake represented exclusion criteria. All subjects received standard clinical care and were followed up for a 3-year period after completing the two examinations. During this follow-up period, patients’ diagnoses, PD or other parkinsonisms, were confirmed.

### 2.1. DAT-SPECT

Patients received activity of 111–185 MBq of 123I-FP-CIT, and SPECT images were acquired approximately 4 h post-injection. Potassium iodide oral solution (Lugol’s solution) or potassium perchlorate 400 mg was orally given 1 h before the tracer injection. A dual-head gamma camera (Infinia, GE Healthcare, Milwaukee, WI, USA) with low-energy high-resolution (LEHR) parallel hole collimators was used. Photopeak energy was centered at 159 keV (±10% wide). The patient’s head was positioned in a head holder. A total of 120 views of 30 s with a view angle of 3 degrees over 360 degrees were acquired on a 128 × 128 matrix, zoom 1.33.

Images were qualitatively evaluated and classified as normal, characterized by physiological uptake of the tracer in both right and left putamen and caudate nuclei, or as pathological in the case of the presence of striatal uptake defects. Semiquantitative data were obtained by calculating the caudate-to-occipital ratio (C/O) and the putamen-to-occipital ratio (P/O) in both basal ganglia, drawing regions of interest (ROIs) on the most representative transversal images.

### 2.2. mIBG-Scintigraphy

For mIBG-SPECT, an activity ranging from 111 to 185 MBq of 123I-mIBG was slowly intravenously injected. Potassium iodide oral solution (Lugol’s solution) or potassium perchlorate 400 mg were orally administered 1 h before the mIBG injection. Acquisitions were performed at 15 min (early) and 4 h (delayed) after the tracer injection with a dual-head gamma camera (Infinia, GE Healthcare, Milwaukee, WI, USA) equipped with LEHR parallel hole collimators and photopeak energy centered at 159 keV (±10% wide). Planar images were acquired for ten minutes with a 128 × 128 matrix, zoom 1. Both early and delayed heart-to-mediastinum ratios (eH/M and lH/M) were calculated as mean count per pixel in the myocardium divided by that in the mediastinum, drawing an ROI on the left ventricle and a 7 × 7 pixel mediastinum ROI in the mid-line upper chest. SPECT cardiac images were acquired immediately after planar images using a 90° rotation, starting at a 45° right-anterior oblique projection and proceeding to the 45° left-posterior oblique projection with a step-and-shoot technique (64 projections, 30 s of duration per frame in non-gated mode). A 64 × 64 matrix was used for SPECT studies, zoom factor 1. SPECT images were reoriented in short, vertical long and horizontal long heart axes after filtered-back-projection (FBP) reconstruction using Myovaton software(GE Healthcare) implemented on a Xeleris Duo platform (GE Healthcare). Heart images were analyzed with a standard 17-segment model. All 17 segments were scored using a 5-point scale where myocardial 123I-mIBG uptake was expressed as a percentage of the maximum myocardial uptake. Defect scores were calculated as the sum of segmental scores, thus obtaining the early and late summed scores (ESS and LSS) [27,28].

### 2.3. Statistical Analysis

The diagnostic odds ratio (OR) with a 95% confidence interval (CI) was evaluated for age, for right C/O, right P/O, left C/O and left P/O derived from DAT-SPECT and for eH/M and lH/M derived from mIBG- SPECT. These were estimated using univariate logistic regression models. The accuracy of each exam was evaluated using receiver operating characteristic (ROC) curves and the related area under the curve (AUC).

## 3. Results

All subjects (n = 288) were de novo, drug-naïve patients showing symptoms suggestive of possible PD or parkinsonism non-PD. Mean age was 66.6 ± 9.9 years, and 173 patients were males. The diagnosis of PD was confirmed in 162 patients (56.2%) after three years of clinical follow-up, while 126 patients (43.8%) received a diagnosis of non-PD. Baseline patients’ characteristics are shown in Table 1.

The univariate analysis including age and both mIBG-SPECT and DAT-SPECT parameters showed that the only significant values for predicting PD in our population were eH/M, lH/M, ESS and LSS obtained from mIBG-scintigraphy (*p* < 0.001) (See Table 2).

Concerning ROC curves, both eH/M and lH/M showed good (>90%) area under the curve values (AUC = 0.918 and AUC = 0.938, respectively), reflecting high sensitivity and specificity values in this population of PD patients.

**Figure 1:**
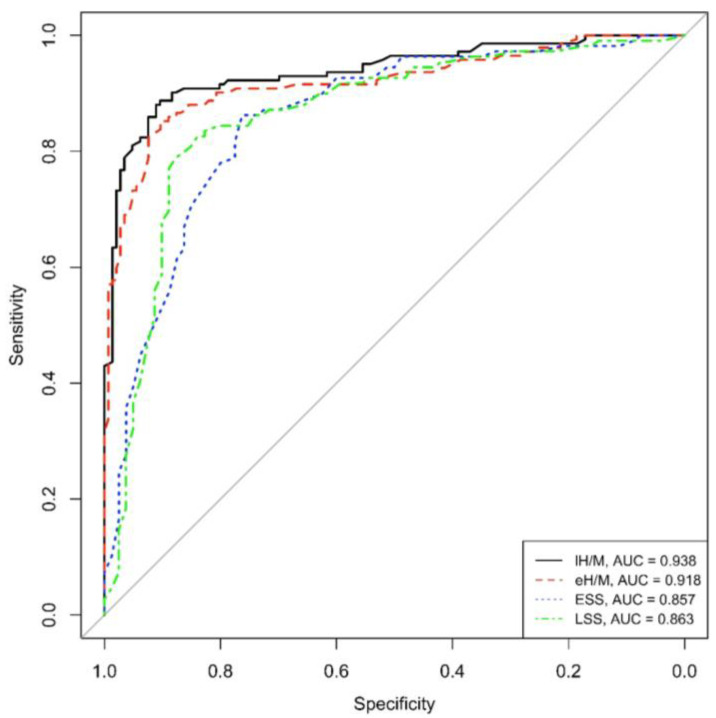
ROC eH/M, lH/M, ESS and LSS (eH/M: early heart-to-mediastinum ratio, lH/M: delayed heart-to-mediastinum ratio, ESS: early summed score, LSS: late summed score), AUC: area under the curve.

Similarly to mIBG-scintigraphy planar parameters, both ESS and LSS obtained from tomographic imaging revealed an optimal AUC, specifically AUC = 0.857 for ESS and AUC = 0.863 for LSS, in our PD population (See Figure 1).

**Figure 2:**
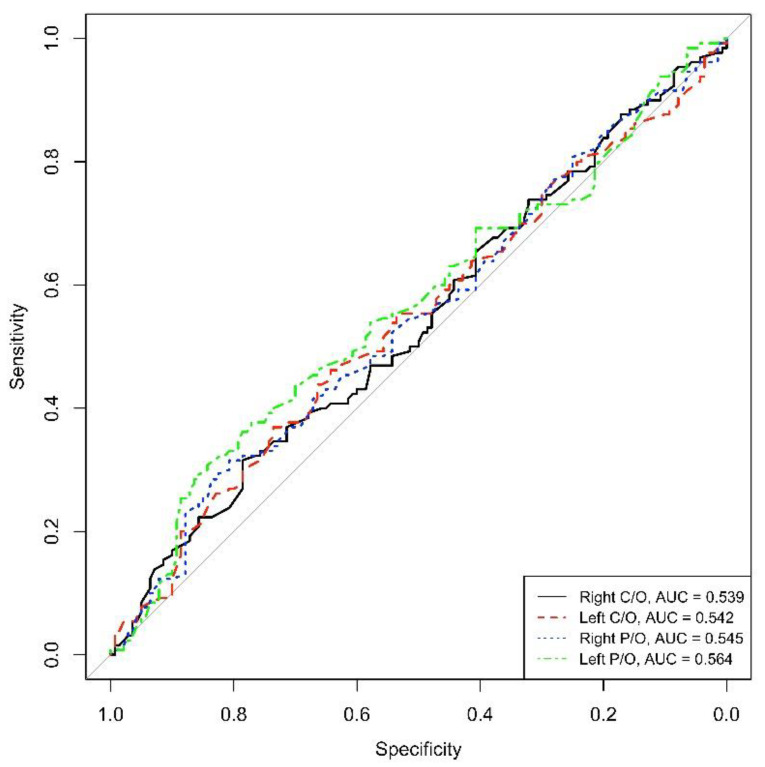
ROC right and left C/O, right and left P/O (C/O: caudate-to-occipital ratio, P/O: putamen-to-occipital ratio), AUC: area under the curve.

On the contrary, the AUC calculated for the DAT-SPECT parameters did not reveal high values of sensitivity and specificity for the diagnosis of PD in those de novo patients (AUC = 0.539 for right C/O, AUC = 0.542 for left C/O, AUC = 0.545 for right P/O and AUC = 0.564 for left P/O) (See Figure 2).

## 4. Discussion

### 4.1. Clinical Criteria

Excluding non-degenerative forms, the principal causes of parkinsonism are PD and atypical parkinsonisms such as PSP, MSA, LBD and CBS. Particularly challenging for the clinician given the broad phenotypic overlap, especially in the early stages of disease, is the differential diagnosis between PD and some atypical degenerative parkinsonisms. For example, you can cite PSP-RS as clinically related with significant oculomotor dysfunction and postural instability, which represents the most common PSP phenotype [24], or PSP-P, diagnosed in 14–35% cases of PSP [29] and associated with a less severe course of disease, often with asymmetric onset, tremor and moderate initial response to Levodopa, and, furthermore, the MSA-P variant, which presents rapidly progressive parkinsonism, postural instability within 3 years of motor onset and poor response to Levodopa [26]. As already mentioned, non-PD was diagnosed in patients with clinical criteria of PSP [23,24], LBD [25] and MSA [26]. PSP, also known as Steele Richardson Olszewski syndrome, is a chronic neurodegenerative condition that affects only a small portion of the brain. It has an impact on your walking, thinking, swallowing and eye movement. There may be more symptoms as well. “Progressive” refers to a situation where the symptoms of the underlying neurodegenerative disorder worsen over time. As “supranuclear” refers to injury above the brain regions responsible for moving your eyes, “palsy” refers to the inability to move your eyes. “Palsy” is a term for muscle weakness or dysfunction. There are numerous signs of Richardson syndrome, some of which include problems with balance and walking, abnormal language, issues with thinking and memory, difficulty controlling eye movement is difficult especially while gazing down, a face with big eyes and a blank expression [30,31]. Progressive dementias like LBD affect thinking, reasoning and independent function. REM sleep behavior disorder, recurring visual hallucinations, spontaneous shifts in attention and alertness, slow movement, tremors and rigidity are some of its characteristics. Along with other types of dementia, including Alzheimer’s disease and vascular dementia, LBD is one of the causes of dementia. LBD primary signs and symptoms include alterations in logic and thinking, erratic cognition with delirium-like symptoms, well-formed visual hallucinations that recur frequently, REM sleep behavior disorder characterized by dream acting, spontaneous parkinsonism accompanied by rigidity, rest tremors and slowness of movement. Other signs can include difficulty understanding visual information; malfunctions of the “automatic” (autonomic) nerve system, which regulates bodily activities such perspiration, blood pressure, heart rate, digestion and libido; and possible serious memory loss that is less obvious than in Alzheimer’s disease [32,33]. MSA is a neurodegenerative disorder that affects both the autonomic nervous system, which regulates automatic bodily processes like blood pressure and digestion, and the central nervous system, which controls how a person moves. Shy-Drager syndrome, olivopontocerebellar atrophy (OCPA) and striatonigral degeneration were the previous names for MSA. The progressive loss of function and death of several types of nerve cells in the brain and spinal cord are reflected in the symptoms of MSA. One of the neurological conditions classified as an atypical parkinsonian disorder is MSA. The early signs of the condition, which might include stiffness, tremor and slowness of movement, can make it difficult to differentiate them from those of Parkinson’s disease. Initial signs may include stiffness, tremor, and slowness of movement, clumsiness or an inability to coordinate, squeaky, trembling voice, stumbling or dizziness and issues with bladder control. The first signs of the condition typically occur in a person’s 50s and worsen over the course of five to ten years. A person with MSA will find it harder to move around and will finally become bedridden. In the later stages of the disease, MSA patients frequently experience swallowing issues that can result in pneumonia [34,35].

### 4.2. Functional Neuroimaging

Improving diagnostic accuracy is crucial, as both prognosis and response to levodopa are different for PD and non-PD. The functional neuroimaging nuclear medicine techniques mainly used in clinical practice are represented by 123I-FP-CIT and 123I-mIBG-scintigraphy [36]. It is well known that 123I-FP-CIT represents a sensitive method for detecting presynaptic DA degeneration. As it is necessary to have a decline of approximately 50–60% of striatal DA before typical motor symptoms appear, it seems unlikely that genuine PD patients can have normal DA transporter function. These observations have led to studies that demonstrated that subjects with normal DA transporter imaging either have been misdiagnosed (non-degenerative conditions mimicking PD such as dystonia, essential tremors or metabolic causes) or have resulted as positive in a second, follow-up DAT-SPECT [37]. Despite the good sensitivity of DAT-SPECT, its diagnostic value is limited by several issues, including the inability to differentiate PD from other parkinsonian syndromes associated with striatal DA deficiency. Myocardial scintigraphy with 123I-mIBG can be used to assess the integrity of postganglionic sympathetic fibers distinguishing PD from non-PD. Recently, Treglia et al. performed a meta-analysis on the accuracy of mIBG-scintigraphy, confirming the high sensibility and specificity of the exam: the pooled sensitivity of mIBG scintigraphy in detecting PD was 88%, and the pooled specificity of mIBG scintigraphy in discriminating between PD and non-PD was 85% [38]. Similarly, Orimo et al. analyzed 13 studies confirming high sensibility and specificity, in particular of lH/M. Although patients with mild Hoehn-Yahr stage or short-duration disease may have myocardial mIBG abnormality less frequently, this review confirmed a sensitivity of 94.1% and a specificity of 80.2% for lH/M even when PD is limited to early stage (Hoehn-Yahr stage one or two) [39]. Pathology and imaging studies have shown that patients with PD have a prolonged period of neuronal degeneration, in which typical motor symptoms have not yet developed and non-motor ones such as depression, sleep disorders, constipation and impaired olfaction may represent the only clinical manifestations [40]. Since non-motor symptoms have proven to be accompanied by low mIBG uptake [41], mIBG-scintigraphy can be a useful marker during the DAT-SPECT negative period to identify premotor PD [42].This multicenter study confirmed the superior diagnostic accuracy of the mIBG exam compared to DAT-SPECT in diagnosing PD in de novo patients, probably related to the fact that damage to the presynaptic postganglionic sympathetic fibers occurs earlier than damage to the presynaptic terminals of dopaminergic neurons. Consequently, it does not seem mandatory to combine both the exams in this stage of the disease, as mIBG-scintigraphy alone is adequate for diagnosis. According to the literature, lH/M confirmed a very high diagnostic accuracy, but eH/M was shown to have similar values of sensitivity and specificity. These considerations are in line with the results reported in a previous paper demonstrating the high diagnostic value of eH/M in PD patients [26]. In our multicenter study, we proved how in addition to planar mIBG-scintigraphy parameters, both ESS and LSS obtained from tomographic imaging present adequate values of sensitivity and specificity in our setting of patients. Considering the high diagnostic accuracy of both eH/M and ESS, we can assume that images obtained at 15 min after tracer administration may be sufficient for PD diagnosis. In a recent study, the authors interestingly proposed a shorter protocol for mIBG-scintigraphy based on a single immediate planar acquisition performed at 5 min after injection and demonstrated its diagnostic accuracy in PD patients [43]. Such an assumption may have important implications, as faster acquisition times could improve patients’ compliance with the exam, avoiding the long waiting times required for late images. Moreover, such findings may lead to a simplification of mIBG-scintigraphy acquisition protocols in the case of diagnostic early acquisitions.

## 5. Limitations

A mention of some major limitations and drawbacks of this study is needed. First of all, despite the study being a multicentric study involving two different Italian centers, the number of patients with diagnosis of PD is relatively low given the inclusion criteria of being de novo, drug-naive patients and having undergone both DAT-SPECT and mIBG-scintigraphy within one month of each other. In addition, with respect to the male population, the female one was less represented. With regard to differential diagnosis with PD, it is worth highlighting that differentiating PD from PSP, MSA and LBD, although clinically relevant, was considered beyond the scope of this work, and, consequently, all these entities were overall referred to as “non-PD.” To conclude, the lack of availability of mIBG-scintigraphy represents one of the major concerns regarding the clinical application of this imaging technique, despite its proven role in the diagnostic framework of neurodegenerative disorders.

## 6. Conclusions

Given the challenges in both PD diagnosis and differential diagnosis with non-PD, exploring the full potential of available functional imaging modalities could further help the clinician in this challenging task. The results of this multicentric study show that mIBG-scintigraphy has higher diagnostic accuracy than DAT-SPECT in de novo PD patients, indicating how the superiority of the heart study could make the combined use of both exams not mandatory in this phase of the disease. Despite all mIBG-scintigraphy parameters revealing high diagnostic value in this setting of patients, the high diagnostic value of both planar and tomographic early parameters is especially valuable, potentially resulting in shorter acquisition times without compromising the diagnostic value but rather simplifying acquisition protocols and improving patients’ compliance.

## Figures and Tables

**Table 1:** Baseline patients’ characteristics.

Baseline Characteristics	Patients (n = 288)	%
Mean age	66 ± 9.8 years	
Sex		
Male	173	60.0%
Female	115	40.0%
Diagnosis		
PD *	162	56.2%
non-PD *	126	43.8%

* Parkinson’s disease (PD).

**Table 2:** Univariate analysis of age, mIBG-SPECT and DAT-SPECT parameters.

Variable	OR	Low IC	Up IC	*p*-Value
Age	1.009	0.985	1.033	0.461
eH/M	0.240	0.207	0.289	<0.001
lH/M	0.310	0.277	0.355	<0.001
ESS	1.020	1.014	1.020	<0.001
LSS	1.020	1.013	1.018	<0.001
Right C/O	0.977	0.926	1.022	0.275
Right P/O	0.960	0.915	1.017	0.185
Left C/O	0.970	0.921	1.019	0.220
Left P/O	0.950	0.903	1.003	0.068

eH/M: early heart-to-mediastinum ratio, lH/M: delayed heart-to-mediastinum ratio, ESS: early summed score, LSS: late summed score, ROC right and left C/O, right and left P/O (C/O: caudate-to-occipital ratio, P/O: putamen-to-occipital ratio).

## Data Availability

Not applicable.

## List of Abbreviations:

PD: Parkinson’s disease; SPECT: single-photon emission computed tomography; DAT: dopamine transporter; non-PD: neurodegenerative parkinsonian syndromes; PSP: progressive supranu-clear palsy; MSA: multiple system atrophy; CBS: corticobasal syndrome; mIBG-scintigraphy: 123I-labeled guanethidine analog “metaiodobenzylguanidine”; eH/M: early heart-to-mediastinum ratio, lH/M: delayed heart-to-mediastinum ratio, ESS: early summed score, LSS: late summed score, ROC right and left C/O, right and left P/O (C/O: caudate-to-occipital ratio, P/O: putamen-to-occipital ratio); AUC: area under the curve.
